# An empirical approach to the definition of the target margins in eye radiosurgery

**DOI:** 10.1002/acm2.13982

**Published:** 2023-07-03

**Authors:** Franco Perrone, Concetta Laliscia, Antonio C. Traino, Maria Tripodi, Federica Cresti, Francesca Guido, Federica Genovesi Ebert

**Affiliations:** ^1^ Health Physics Division Azienda Ospedaliero Universitaria Pisana Pisa Italy; ^2^ Radiation Oncology Division Azienda Ospedaliero Universitaria Pisana Pisa Italy; ^3^ Casa di Cura “S. Rossore,” Radiation Oncology Unit viale delle Cascine Pisa Italy; ^4^ Ophthalmic Surgery Division Azienda Ospedaliero Universitaria Pisana Pisa Italy

**Keywords:** CBCT, eye tracking, planning target volume, stereotactic radiosurgery, uveal melanoma

## Abstract

**Introduction:**

A system for stabilizing and monitoring eye movements during LINAC‐based photon beam one single fraction stereotactic radiotherapy was developed at our Institution. This study aimed to describe the feasibility and the efficacy of our noninvasive optical localization system that was developed, tested, and applied in 20 patients treated for uveal melanoma.

**Methods:**

Our system consisted of a customized thermoplastic mask to immobilize the head, a gaze fixation LED, and a digital micro‐camera. The localization procedure, which required the active collaboration of the patient, served to monitor the eye movements during all phases of the treatment, starting from the planning computed tomography up to the administration of radiotherapy, and allowed the operators to suspend the procedure and to interact with the patient in case of large movements of the pupil.

**Results:**

Twenty patients were treated with stereotactic radiosurgery (27 Gy in one fraction) for primary uveal melanoma. All patients showed a good tolerance to the treatment; until now, all patients were in local control during the follow up and one died for distant progression 6 months after radiosurgery.

**Conclusions:**

This study showed that this noninvasive technique, based on eye position control, is appropriate and can contribute to the success of LINAC‐based stereotactic radiotherapy. A millimetric safety margin to the clinical target volume was adequate to take account for the organ movement. All patients treated till now showed a good local control; failures in the disease control were due to metastatic spread.

## 1 INTRODUCTION

Uveal melanoma (UM) is an aggressive primary malignancy that, if not treated, tends to infiltrate surrounding tissues and to metastasize in other organs.^1^ Until the 80s, enucleation was considered the elective treatment of UM. Over the years, various globe‐sparing approaches have been defined; although enucleation remains an appropriate treatment in relation to the quoad vitam prognosis, the prospective North American COMS multicenter study demonstrated that a radiotherapy approach focused on the local disease control can be administered without reducing patient survival.^2^

Possible conservative treatments for ocular tumors include brachytherapy,^3^, ^4^ proton beam therapy,^5^ radiosurgery using GammaKnife^6^ or CyberKnife,^7^ and LINAC stereotactic radiotherapy (SRT).^8^ These therapeutic strategies aim at administering in one single fraction or at most in 3−5 fractions a radical dose to a very low target volume (0.3−5 cm^3^). External beam radiotherapy (EBRT) is generally adopted in patients unsuitable for plaque brachytherapy (large and/or posterior lesions).

A very challenging task for the team is to assess the appropriate margins to the clinical volume. The use of localization and immobilization techniques together with eye monitoring helps to setup narrow tolerance margins, as it allows obtaining the local disease control minimizing the morbidity. Late toxicities related to eye irradiation are a consequence of vasculopathy induced by the radiation spreading outside of the target volume: most common effects include radiation retinopathy, glaucoma, and cataract.

Together with traditional systems of immobilization of the patient's head (thermoplastic mask, stereotactic helmet, or other), it is also essential to adopt specific devices to stabilize and to monitor the eye. Some Authors have developed and described invasive technical solutions suitable for the purpose, exploiting blepharostats, corneal suction cups, sutures of the ocular muscles, peri‐bulbar anesthesia and scleral coils, but noninvasive devices based on a fixation light and a monitoring camera appear to be the most viable and promising solutions.^9^, ^10^ In 2003, a Vienna group^11^, ^12^ proposed a system suitable for treating UMs with LINAC in collaborating patients not suitable for brachytherapy and with an acceptable residual visual acuity. This system consisted of a rigid assembly composed of a stereotactic mask, a plastic tube, a mirror, a gaze fixation light, and a micro‐camera.

Similar systems were described in the following years by some groups of the University Hospital of Toronto,^13^ Montréal,^14^, ^15^ Melbourne,^16^ Osaka,^17^ and San Francisco,^18^ respectively: the proposed devices were slightly different among them and were applied to SRT both with LINAC and Cyberknife; the Authors claimed reproducibilities ranging from 0.3 to 2 mm.

The aim of our work is to present a noninvasive system for controlling and recording eye movements during SRT for UM. Our system was composed of a customized thermoplastic mask for SRT and an adjustable blinking LED for controlling patient's gaze; the mask and the LED were both rigidly fixed to the treatment couch. A computerized micro‐camera (also attached to the couch), was used to display the eye on‐line on a notebook and to record its movements during the whole treatment, that is, planning Computed Tomography (CT) scan, Cone Beam CT (CBCT) verification, and Stereotactic Radiosurgery (SRS) treatment session. Basing on the analysis of the eye movements during the CT scan, an additional margin value was chosen to be added to the Clinical Target Volume (CTV)^19^ during the delineation and planning phase: the greater is the patient's ability to collaborate, the smaller is the expansion of the CTV to Planned Target Volume (PTV) and, therefore, the risks of target under‐dosage or radio‐induced toxicity are minimized.

At the treatment time, the patient was positioned to the LINAC and the fixation light and the camera were mounted reproducing the CT set‐up. After checking the setup with a cone beam CT, the initial positioning of the eye was verified on the laptop monitor. Finally, the dose was administered while the eye was continuously monitored.

The purpose of this study was to assess the effectiveness and the accuracy of the proposed system and to confirm that the applied treatment margins were clinically acceptable.

## 2 METHODS

An EBRT is indicated in patients with large and/or posterior UM, not suitable for brachytherapy: in these cases, the therapeutic success depends on a satisfactory dosimetric coverage of the lesions, pursued by reducing all possible uncertainties in the irradiation. In case of very small irradiation volumes, SRS could be administered in a single fraction.

The system adopted in our center to localize the lesion and “freeze” the eye movements consisted of a thermoplastic stereotactic mask to immobilize the skull on a carbon fiber board (ConBine, Candor, DK) and an optical system composed of a digital micro‐camera, a blinking red LED with an anisotropic forward pattern of emission, a plastic mirror, and an adjustable arm also fixed to the board. The arm was used to support and individually regulate the optical components. The board could be locked both on the CT table and on the LINAC couch. A hole was made on the mask to uncover the eye; the camera and the LED were oriented in the cranial‐caudal direction at the level of the upper dental arch. Some fiducial markers on the mask were used to facilitate the camera repositioning and orientation. The mirror was used to obtain an isometric frontal view of the eye with the camera and to allow the patients fixing the LED light.

Roughly speaking, the eye globe can be approximated by a rigid sphere rotating around its center in the socket: since the iris and the tumor hold their relative positions during the treatment, they are subject to identical angular displacements. The camera could record only eye movements in the medio‐lateral and cranial‐caudal direction, whereas the AP displacements were neglected.

The camera captured snapshot images or video sequences of 640 × 480 pixels with an acquisition rate of 33 frames/s; it was equipped with a cable for power supply and data transmission via an USB connector. The spatial resolution reached at the focal distance of 150 mm was 0.075 mm. The images produced were viewed online and recorded on a computer in AVI format. Video recording was started and stopped manually so to be synchronous with the radiological exposures. The Personnel in the control room carefully watched the eye movements on‐line; the video sequences were saved and subsequently analyzed off‐line.

The acquired images and video sequences were calibrated on the plan of the pupil with a ruler. For each patient, a two‐dimensional Cartesian frame was defined, with its origin in the center of the pupil while fixing the LED (chosen as the Reference Origin), the x in the LR direction and the y along the SI direction.

The planning CT scans (Discovery RT CT scanner, GE Healthcare, USA) with intravenous injection of contrast agent were performed with 0.625 mm slice thickness from the apex to the base of the skull. Images were reconstructed with a matrix of 512 × 512 pixels and a field of view of 300 mm. The upper resolution limit for CT simulation and contouring was assumed to be about 1.0 mm in all directions.

A snapshot view taken before CT scan with the initial eye's position was stored as the reference image.

Treatment planning was prepared using Eclipse 16.1 with the Acuros XB algorithm (Varian Medical Systems, Palo Alto, California, USA). The radiation oncologist drew the contours of CTV, also based on other available diagnostic exams (fundus exams, US, MRI) and healthy structures at risk (i.e., optic nerves, macula, anterior chambers, lachrymal glands, lenses, whole counter‐lateral eye, and chiasm). An expansion of few millimeters of CTV to PTV was applied to account for uncertainties in simulation, planning, localization, and treatment.

All treatments were delivered using a True Beam LINAC (Varian Medical Systems, Palo Alto, California, USA) with Flattening Filter Free (FFF) beams (maximum dose rate was 1400 MU/min) and Rapidarc (Varian Medical Systems, Palo Alto, California, USA) Volumetric Modulated Arc Therapy (VMAT) technique. The LINAC is equipped with a HD120 multi‐leaf collimator having 2.5 mm wide leaves in its central section. The mechanical displacement of the LINAC radiological isocenter and its position reported by the CBCT system was within ± 0.5 mm. The quality control checks showed that the mechanical isocenter reproducibility was ± 0.5 mm.

Rapidarc treatments were configured with two to three arcs. A dose of 27 Gy in one single fraction was prescribed with the PTV(95%) = 95% dose‐volume constraint.

At the beginning of the treatment sessions, the optical components for eye monitoring were repositioned and checked with rulers and a kVolt Cone Beam Computed Tomography (CBCT) was acquired with the LINAC imaging system and compared with the planning CT in order to check the patient's alignment. Corrections could be applied with the Varian PerfectPitch 6D couch (Varian Medical Systems, Palo Alto, California, USA) for the best repositioning of the bony structures and of the body profile. The quality assurance tests performed on our couch evidenced a setup accuracy of less than 0.2 mm in linear movements (X‐Y‐Z of the couch frame of reference) and less than 1.1° in angular displacements (pitch‐roll‐yaw), similar to the findings of other Authors.^20^

Then, a new snapshot was acquired with a ruler positioned near the pupil to calibrate the scale of distance on the next video recordings and to reposition the pupil in the Origin. The patients were carefully instructed to gaze the LED light. A video was acquired during the CBCT and the VMAT treatment. Some reference markers were reported on the control monitor to quantify the eye shifts: in case of large displacements, the treatment could be suspended and the patient could be solicited to focalize on the LED.

Video analysis was performed using Tracker, an open source educational tool for physics built on java platform (Open Source Physics by Douglas Brown).^21^ Tracker was used to mark the position of the center of the pupil in each video frame. Under favorable ambient light conditions, an automatic (operator‐assisted) tracking of the eye was achieved in the majority of the video sequences; in the remaining frames, the eye tracking was carried out manually. The length scale could be calibrated and a couple of orthogonal axes could be fixed, allowing to determine the (x_i_; y_i_) coordinates of the center of the pupil from the i‐th frame. The sequences in which the patients blinked were excluded from the analysis.

## 3 RESULTS

All the 20 patients recruited for this study were in good general conditions with satisfactory visual acuity and they were able to maintain the gaze. In patients with poor visual acuity in the affected eye, the monitoring set‐up was mounted to observe the contra‐lateral eye.

The LINAC couch could be displaced on six degrees of freedom to correct the misalignments evidenced by the CBCT: applied translations were within 2 mm (average values, −0.18 , −0.14, and −0.35 mm in the vertical, longitudinal, and lateral directions, respectively) and angles of rotation were less than 2° (average values, 0.5 °, 0.1 °, and −0.4 ° for pitch, roll, and rotation, respectively).

In all the videos, the possible movements of the mask were carefully checked by tracking one of its fiducial markers. The maximum excursion of the mask was about 0.35 mm, and it was due to the respiratory movements; this value was considered as the best accuracy attainable with the mask mounting.

The duration of all video sequences was between 15 and 217 s (i.e., 400−7200 frames). The mean duration of CT and CBCT video recordings were 16 s (range, 15−21 s) and 20 s (range, 19−21 s), respectively. The mean duration of SRS video sessions was 155 s (range, 92−217 s). The percent of frames excluded from the analysis was on average 5% (mainly the images of eye blinking) and in all cases they resulted to be less than 10% of each video.

The (x_i_; y_i_) couples of coordinates of the center of the pupil were extracted from the i‐th frame along the x (LR) and y (SI). About 70% of the pupil's positions were detected automatically.

**FIGURE 1:**
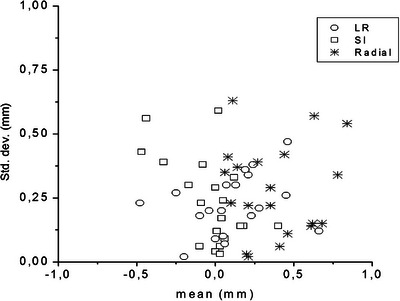
Displacements per patients in CT. Patient‐specific mean and standard deviation of displacements during CT examination. Circles: left‐right direction; squares: superior‐inferior direction; asterisks: radial shifts from origin.

If N indicated the number of frames of a video, the mean values X_m_=Σ_i_ (x_i_)/N and Y_m_=Σ_i_ (y_i_)/N, respectively, and the standard deviations σ_X_=(Σ_i_ (x_i_−X_m_)^2^/N)^1/2^and σ_Y_=(Σ_i_ (y_i_‐Y_m_)^2^/N)^1/2^, respectively, were calculated. For each frame we calculated the radial pupil displacements r_i_=(x_i_^2^ + y_i_^2^)^1/2^from the 0rigin, together with their mean value R_m_=Σ_i_ (r_i_)/N, their standard deviation σ_R_=(Σ_i_ (r_i_‐R_m_)^2^/N)^1/2^, the maximum value R_max_ of the r_i_ and the percent fractions ΔR_1_ and ΔR_2_ of frames with r_i_≤ 1 and 2 mm, respectively. Figure 1 shows the individual specific mean values and their respective standard deviations of displacements in LR, SI and radial directions in CT videos. The CT video statistics were used to decide the appropriate margin CTV‐PTV taking account of the compliance of each patient to the eye immobilizing system.

A number of Authors gave an explicit formula to determine the appropriate set‐up margins in radiotherapy.^22^, ^23^, ^24^, ^25^, ^26^ Each formula was built for specific situations of fractionations, beam profiles, target shapes, sites of irradiation, biological dose equivalence, or radiation techniques. Margins should take account of systematic uncertainties, as well as of random uncertainties in the positioning of the patient. Moreover, it has been shown that the degrees of freedom of the localization systems could change the relative weight of the errors components.^22^

The 3D systematic sources of error of our site include the isocenter localization (± 0.5 mm), the CBCT reproducibility (± 0.5 mm), the couch accuracy (± 0.2 mm), and the mask reproducibility (± 0.35 mm): the combined 1σ 3D systematic error was therefore Σ_3_ = 0.81 mm. Although the angular accuracy of the couch could probably contribute to Σ_3_, its effect on coplanar arcs was quite complex to be quantified.

As X_m_ and Y_m_ could be considered as two‐dimensional positional offsets, the 2D contribution to the systematic uncertainty for each patient was estimated from CT as Σ_2_ = (X_m_^2^ + Y_m_^2^)^1/2^.

Random errors during SRS could be evaluated in a number of ways: in this study we assumed that each patient tended to have a similar behavior during all the phases of the treatment and, therefore, random uncertainty was assumed to be the CT σ_R_.

**FIGURE 2:**
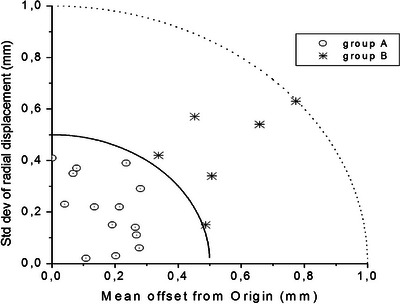
Systematic and random errors per patient in CT. Patient‐specific mean offset Σ_2_ and radial standard deviation σ_R_ during CT examination. Dotted circles: group A; asterisks: group B; continuous line: (Σ_2_^2^+σ_R_^2^)^1/2^ = 0.5 mm; dotted line: (Σ_2_^2^+σ_R_^2^)^1/2^ = 1.0 mm.

The CT eyes displacements were very small, with all the Σ_2_ ≤0.77 mm (|X_m_|≤0.66 mm in LR and |Y_m_|≤0.48 mm in SI) and the σ_R_ ≤0.63 mm. Mean values of ΔR_1_ and ΔR_2_ were 97% (range, 50−100%) and 100% (range, 99−100%), respectively. The CT σ_R_ are shown in Figure 2 as a function of the respective Σ_2_. The mean values of Σ_2_ and σ_R_ on the whole sample were 0.28 mm (std. deviation, 0.21 mm) and 0.28 mm (std. deviation, 0.18 mm), respectively (see Table 1).

**TABLE 1:** Systematic and random uncertainties of eyes' positions

		CT	CBCT	SRS
Patient's data		Mean ± St. dev. (mm)	Mean ± St. dev. (mm)	Mean ± St. dev. (mm)
A+B (n = 20)	Σ_2_	0.28 ± 0.21	0.34 ± 0.30	0.29 ± 0.19
	σ_R_	0.28 ± 0.18	0.47 ± 0.24	0.26 ± 0.14
A (n = 14)	Σ_2_	0.17 ± 0.10	0.37 ± 0.31	0.28 ± 0.19
	σ_R_	0.21 ± 0.13	0.49 ± 0.27	0.21 ± 0.12
B (n = 6)	Σ_2_	0.54 ± 0.18	0.26 ± 0.27	0.31 ± ± 0.23
	σ_R_	0.44 ± 0.26	0.44 ± 0.17	0.39 ± 0.11

Mean offsets and respective standard deviations. A and B are two subgroups of patients, Σ_2_ and σ_R_ are the two‐dimensional systematic and random errors, CT is the planning computed tomography, CBCT is the Cone Beam CT, and SRS is the stereotactic radiosurgery.

We observed that the combinations in quadrature of Σ_2_ and σ_R_ of each patient could be collected in two groups, that is, those with (Σ_2_^2^+σ_R_^2^)^1/2^ ≤0.5 mm (group A, 14 patients) and those with 0.5 mm < (Σ_2_^2^+σ_R_^2^)^1/2^ < 1.0 mm (group B, six patients), respectively, as shown in Figure 2 and reported in Table 1. Moreover, the mean values of ΔR_1_ were 86% (range, 50−100%) and 100% (range, 99−100%) for group A and B, respectively, whereas ΔR_2_ were substantially 100% for both groups. A one‐tailed Mann‐Whitney test showed that the two groups of patients were significantly different at a level of *P* = 0.00031 (z‐score was −3.42286) for Σ_2_ and *P* = 0.0084 for σ_R_ (z‐score was −2.39188), respectively.

For each group of patients, Σ_3_ and Σ_2_ were added together and then were combined in quadrature with σ_R_ to give global uncertainties of 1.04 mm (group A) and 1.42 mm (group B), respectively. Finally, we determined the margin CTV‐PTV by doubling these values (and approximating to the nearest integer value) to take account of the resolution limits of our apparatus and of the unaccounted uncertainties: applied margins were 2 mm for group A and 3 mm for group B, respectively (Table 2).

**TABLE 2:** CTV to PTV expansion

	[(Σ_2_+Σ_3_) ^2^ + σ_R_^2^]^1/2^ (mm)	Applied margin (mm)	Van Herk margin (mm)
A+B (n = 20)	1.13	–	3.13
A (n = 14)	1.00	2	2.77
B (n = 6)	1.42	3	3.95

Mean uncertainties on the target localizations and CTV to PTV margins. A and B are two subgroups of patients, Σ_2_ and σ_R_ are the 2‐dimensional systematic and random errors in the video recordings, Σ_3_ is the three‐dimensional systematic setup error.

Referring to the Van Herk formalism,^22^ we calculated the margin CTV‐PTV as follows:

(1) $$\mathrm{PTV}\mathrm{MARGIN}\approx 2.55\;\sum_{3}+2.15\;\sum_{2}+1.64\;\sigma_{R},$$

that is, the CTV expansion ensuring the dose coverage of the lesion with at least the 95% of the prescribed dose in the 90% of the patients; the PTV uncertainty was considered as a combination of two‐ and three‐dimensional systematic sources of error, as well as of random errors.

The Van Herk margin resulted to be approximately 2.9 mm for group A and 3.9 mm for group B: these values are slightly higher than our empirical evaluation but coherent with them. Although the original Van Herk formula was built for a particular case of fractionation scheme, beams shape, Gaussian penumbra, constraints on organs at risk and biological equivalent dose, it can be considered as a superior but significant limit to the appropriate value for our case.

**FIGURE 3:**
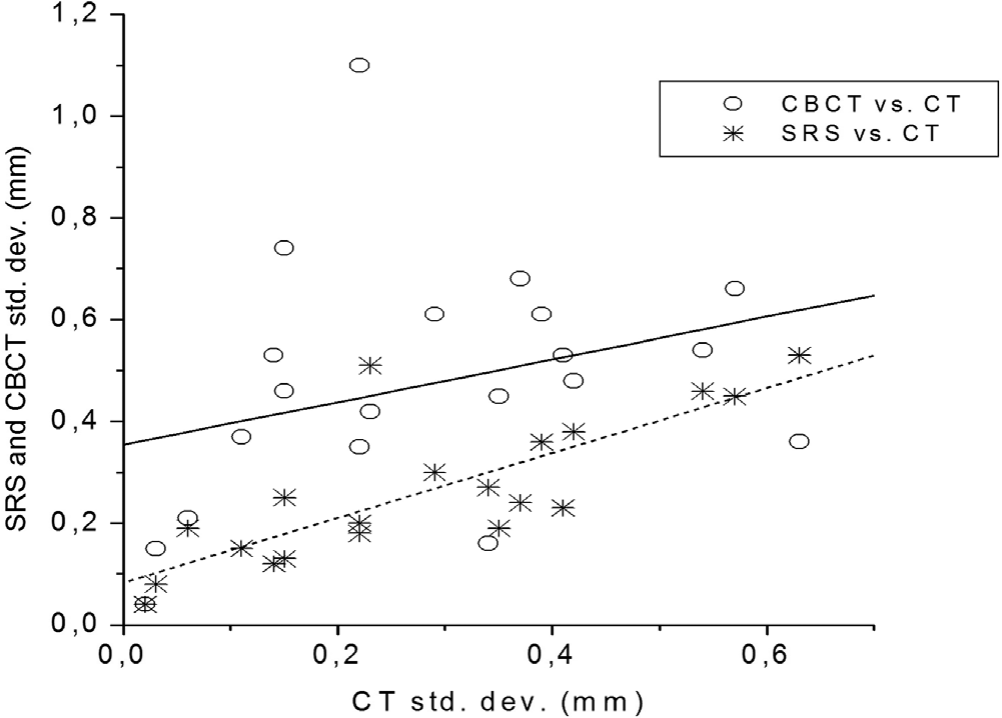
Linear fit of random errors in CBCT and SRS. Patient‐specific σ_R_ for CBCT (circles) and SRS (asterisks) as a function of the respective CT σ_R_, with linear regressions curves of CBCT versus CT (continuous) and SRS versus CT (dotted).

The Σ_2_ and σ_R_ values of CBCT videos were generally larger than those of other series of videos, whereas SRS values were quite similar to those of the CT (Table 1). To assess a possible correlation with the data of the CT videos, both the series were fitted with a linear regression: results are summarized in Table 3 and Figure 3. The ANOVA F‐test gave *P* = 0.179 for CBCT versus CT fit and *P* < 0.0001 for SRS versus CT fit.

**TABLE 3:** Liner fittings of CBCT and SRS random uncertainties

Video sequence	Parameter	Value	Error	t‐test Value	*P* for t‐test	*R*‐square
CBCT	Y axis intercept	0.354 mm	0.099 mm	3.568	**0.0022**	0.098
	Slope	0.419	0.300	1.400	0.179	
SRS	Y axis intercept	0.083 mm	0.037 mm	2.219	**0.040**	0.641
	Slope	0.639	0.113	5.677	**<0.0001**	

Results of linear regression of CBCT σ_R_ versus CT σ_R_ (top) and SRS σ_R_ versus CT σ_R_ (bottom). The table reports the t‐test values for the fitted coefficients of the regression, the corresponding *P* values, and the coefficient of determination R‐square of the fit.

**FIGURE 4:**
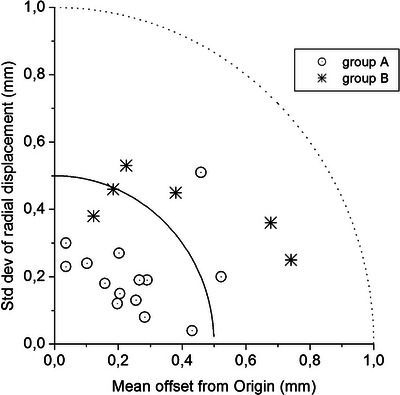
Systematic and random errors per patient in SRS. Patient‐specific offset Σ_2_ and radial standard deviation σ_R_ during SRS. Dotted circles: group A; asterisks: group B; continuous line: (Σ_2_^2^+σ_R_^2^)^1/2^ = 0.5 mm; dotted line: (Σ_2_^2^+σ_R_^2^)^1/2^ = 1.0 mm.

A plot of the SRS individual σ_R_ values as a function of the respective Σ_2_ (see Figure 4) showed a spread of data in fair accordance with that of CT for both groups of patients and, therefore, validated our decisional criterion about the margin.

The irradiation of ocular and peri‐ocular tissues may induce complications; acute lesions may occur within a few weeks after radiotherapy, whereas long‐term adverse events could be delayed from a few months to 1 or 2 years. None of the patients analyzed in the present study experienced acute or late severe toxicity events. The main observed Grade 1−2 acute toxicities were watering eye, photophobia, and conjunctivitis; late Grade 1−2 observed side effects were cataract, exudative retinal detachment, and radiation retinopathy. To date (median follow‐up = 12.8 months; range, 3−20 months), 19 patients were alive in local control and one patient in local control died of distant progression (liver) 6 months after SRS.

## 4 DISCUSSION

The correct positioning of the patient is a key issue for the success of the radiotherapy. In the particular case of SRS on mobile organs, the definition of specific procedures to guarantee adequate levels of accuracy and precision in the administration of the dose is a mandatory step.

This work presents a system based on the design of existing devices adapted to our methodology in order to provide a necessary tool in ophthalmological oncology. The results obtained on 20 patients showed that the device adequately responded to the clinical needs of SRS and could be extended to a larger sample of patients.

Many Authors addressed the issue of the necessary setup margins to the CTV to get a satisfactory coverage of the lesion while minimizing the risk of toxicity.

For multi‐fractionated prostate cancer treatments, Van Herk^22^ showed that systematic and random uncertainties require different setup margins and have a different impact based on the number of degrees of freedom of the possible displacements of the patient. Based on the assumption of a spherical target volume, symmetric traditional photon beams, perfect alignment of the beams to the target and infinite number of fractions, he introduced his well known analytical margin formula that has proven to be also suitable to single fraction regimens with some cautions.

Parker^23^ measured the uncertainties in the multileaf collimator positioning, in CT and MRI spatial localization and fusion and in the relocatable head frame; then, he incorporated these data in a Monte Carlo calculation to evaluate various margins and fractionations. Systematic uncertainties were added linearly and combined in quadrature with random errors (assumed to have a Gaussian distribution).

Zhang^24^ published a margin formula both for 3D and 1D forms, originally intended for single‐fraction frameless SRS and CBCT setup verification, that ensures the prescribed dose to the CTV in 95% of the patients.

Duggar^25^ compared the cited margin formulas with the basic expression of the sum in quadrature of systematic and random errors suggested by ICRU^19^ for SRS with Gamma Knife and CBCT setup verification. He applied the margin recipes to replan 30 previously treated patients and evaluated the target dose coverage and the respect of the normal tissue dose tolerances using a pass/fail scoring system. He found that the Van Herk and the Parker margin formulae were the best candidates to meet both normal tissues and target goals with a CTV‐PTV margin of over 1 mm.

Calvo Ortega^26^ evaluated setup margins in cranial SRS for LINAC treatments guided by CBCT on a skull phantom study. Dosimetric validation of the margins was performed in five real SRS cases: results showed that a 1 mm uniform margin was sufficient to ensure the CTV coverage without compromising the organs‐at‐risk dose tolerances.

Our system based on a light fixation allowed the monitoring of the eye during all the phases of the treatment and maintaining in most cases the eye shifts within 1 mm. We evaluated the extent of the eye movements of each patient with the analysis of videos acquired during CT and then we used these data to define the margin CTV‐PTV for planning.

We guessed that the ability of each patient to control the glaze had an individual behavioral mark in all the phases of the treatment, although anxiety and fear of suffering during the irradiation influenced the capability of fixing the gaze. The CBCT, performed just before the treatment administration, was the first approach to the treatment room: in this phase patients generally exhibited wider eye movements and more frequent blinking, as compared with the CT scan. Afterward, the patients tended to become more confident and, consequently, they exhibited a better compliance during SRS. Our analysis justified our choice to take account of the individual ability to limit the movements, planning with a specific margin for each patient.

## 5 CONCLUSION

To treat large eye tumors, LINAC‐based SRS with a fixation light and a monitoring system represents a feasible, reproducible and accurate option.

Systematic uncertainties in the preparation procedures were < 1 mm and eye movements larger than 1 mm were sporadically observed: therefore, a global margin of 2−3 mm was generally adequate to obtain a good dosimetric CTV coverage in all patients.

Although large UMs are generally associated with a less favorable prognosis, we believe that the satisfactory outcome of this experience is an encouragement to increase the case series and to obtain a longer follow‐up.

## AUTHOR CONTRIBUTIONS

Franco Perrone: conceptual design and practical realization, data collection, data analysis, and text redaction and revision.

Concetta Laliscia: conceptual design, patients' management, CT contouring, and text redaction.

Antonio Claudio Traino: data analysis and critical text revision.

Maria Tripodi: treatment planning, data collection, and critical text revision.

Federica Cresti, Francesca Guido, and Federica Genovesi Ebert: patients' management, data collection, and critical text revision.

## CONFLICT OF INTEREST

The authors declare no conflicts of interest.
